# Iron-Catalyzed C–H Functionalizations under Triazole-Assistance

**DOI:** 10.3390/molecules25081806

**Published:** 2020-04-15

**Authors:** Matteo Lanzi, Gianpiero Cera

**Affiliations:** 1 Laboratoire de Chemie Moléculaire (UMR CNRS 7509), Université de Strasbourg, ECPM 25 Rue Becquerel, 67087 Strasbourg, France; lanzi@unistra.fr; 2 Dipartimento di Scienze Chimiche, della Vita e della Sostenibilità Ambientale, Università di Parma, Parco Area delle Scienze 17/A, I-43124 Parma, Italy

**Keywords:** C‒H activation, iron, 1,2,3-triazole, catalysis

## Abstract

3d transition metals-catalyzed C–H bond functionalizations represent nowadays an important tool in organic synthesis, appearing as the most promising alternative to cross-coupling reactions. Among 3d transition metals, iron found widespread application due to its availability and benign nature, and it was established as an efficient catalyst in organic synthesis. In this context, the use of *ortho*-orientating directing groups (DGs) turned out to be necessary for promoting selective iron-catalyzed C–H functionalization reactions. Very recently, triazoles DGs were demonstrated to be more than an excellent alternative to the commonly employed 8-aminoquinoline (AQ) DG, as a result of their modular synthesis as well as the mild reaction conditions applied for their removal. In addition, their tunable geometry and electronics allowed for new unprecedented reactivities in iron-catalyzed C–H activation methodologies that will be summarized within this review.

## 1. Introduction

Transition metals-catalyzed cross-coupling reactions are a powerful tool in organic synthesis. Since pioneering works in the 1940s [1], and afterwards on modern palladium catalysis [2], they continue to play an important role for the construction of new C–C bonds [3,4]. Despite a wide application in pharmaceutical and agrochemical industries, these reactions require pre-functionalized starting materials and suffer from the production of stochiometric amounts of metal and organic by-products [5]. Therefore, one of the most important goals for the scientific community is to develop new and more suitable synthetic methodologies [6].

In recent years, C–H bond functionalization reactions have emerged as an attractive atom- and step-economical alternative. In this context, precious 4d and 5d metals such as palladium, ruthenium, iridium, and rhodium have been well explored [7,8,9,10]. Albeit the wide applicability and selectivity demonstrated by these metals, their high cost and rather high toxicity constitute a limitation in the development of valuable alternatives to the classic metal-catalyzed cross-couplings [11]. Hence, a more sustainable solution arises from the development of 3d metals-catalyzed methodologies [12,13]. In fact, due to the low toxicity and vast availability, the use of earth-abundant metals such as iron, cobalt, and manganese represents a valuable and appealing area of research [12,13,14,15,16].

Iron is the second most abundant metal on Earth’s crust after aluminum and, therefore, the most abundant transition metal. During life evolution, iron has become essential, and it is present in several functional biological molecules such as cytochrome P450 [17]. The broad catalytic activity of iron in organic synthesis has been well documented [18,19,20,21]. Iron can explore multiple oxidation states (from −2 to +5), rendering its catalytic versatility almost unlimited. Inspired by early reports on cross-coupling reactions [22], the discovery in 2008 of the first example of organometallic iron-catalyzed C–H functionalization [23] constituted an important advance in this area of research, prompting several research groups to investigate in this direction [24,25,26].

It is a matter of fact that the selective activation of an overwhelming number of C–H bonds, present in organic molecules, remains highly challenging. This problem has been mitigated with the introduction of a large number of directing groups (DGs) [27]. These functional moieties, placed in proximity to the C–H bonds are able to coordinate the metal center and guide the substrate functionalization, usually forming thermodynamically stable six- or five-membered metallacycles. DGs are classified as monodentate or bidentate and often characterized by the presence of heteroatoms such as nitrogen, sulfur, or phosphorous, which are strong σ-donors and π-acceptors. Nevertheless, an important point for the application of DGs in organic synthesis can be found in the convenience of the introduction and cleavage of these functional moieties [28,29].

In this context, 8-aminoquinolines (8-AQ) bidentate DGs have proven to be widely applicable for selective *ortho*-C–H activation of benzoic acid derivatives [30,31]. Seminal works reported by Nakamura and co-workers, emphasized the use of the 8-aminoquinoline in C–H bond activation, exploring its use along with iron catalysts [32,33]. However, the limited structural modification, in concomitance with the low chemo-selectivity often resulting from undesired functionalizations of its aromatic ring, restricts somehow the use of 8-AQ as DG in 3d metals-catalyzed C–H activations.

**Scheme 1:**
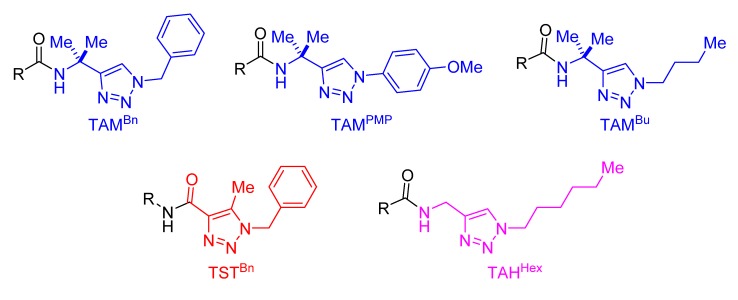
Examples of 1,2,3-triazoles directing groups (DGs).

As a result of longstanding investigations on the use of triazole DGs for C–H functionalizations [34,35], in 2014, Ackermann and co-workers proved the use of a new class of bidentate DGs for ruthenium-catalyzed C–H arylations [36]. The straightforward and modular synthesis of triazoles [37] represents a main advantage that has been explored, parallelly, by other research groups for promoting palladium-catalyzed C–H activation methodologies [38,39]. The copper-catalyzed [3+2] cycloaddition (CuAAC) within an alkyne and an organic azide proceeds under mild reaction conditions to provide 1,2,3-triazoles in excellent yields and complete regioselectivity. As a result, the correct choice of the reaction partners allowed for the synthesis of a broad family of triazoles (Scheme 1).

Importantly, this class of DGs could be easily installed and subsequently cleaved under mild reaction conditions with a wide functional group tolerance, leading to the synthesis of key organic molecules amenable to further structural modifications. From a historical perspective, the first generation of TAM^Bn^ was demonstrated to be a widely applicable bidentate DG for iron-catalyzed C–H transformations. Subsequently, a number of new disconnections could be obtained by a proper interplay of electronic and steric properties of the triazole motif, with the introduction of new families of triazole-based DGs. Within this review, we aim to offer to the reader a complete overview of the synthetic possibility of 1,2,3-triazole directing-groups in the field of iron-catalyzed C–H functionalizations.

## 2. Iron-Catalyzed C–H Arylations

**Scheme 2:**
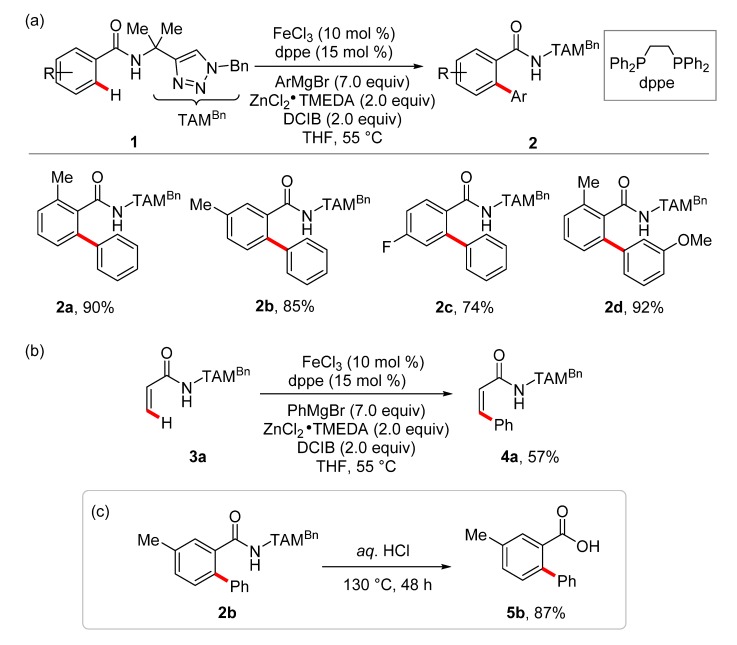
Triazole-assisted C(sp^2^)–H arylation; (**a**) Aryl amides functionalizations; (**b**) Acrylamide functionalization; (**c**) Removal of TAM DG.

After discovering the ability of triazole TAM (triazolyldimethylmethyl), as directing-group for ruthenium-catalyzed C–H arylations of benzamide derivatives, Ackermann and co-workers reported a protocol based on Earth-abundant and non-toxic iron salts [40]. The catalytic system required the use of bidentate phosphine ligands such as 1,2-bis(dipHence, the introduction of a new triazole TAHhenylphosphino)ethane (dppe), an in situ-formed organozinc reagent of type Ar_2_Zn•MgBr_2_(TMEDA), as both base and arylating reagent and an external oxidant such as 1,2-dichloroisobutane (DCIB). This method converted benzamide derivatives **1a-d** in to their corresponding *ortho*-arylated products **2a-d** in good yields and functional group tolerance (Scheme 2a). Interesting, by intermolecular competition experiments, TAM^Bn^ DG was found to be better performing with respect to the so far widely employed 8-aminoquinoline (AQ). It is noteworthy that the catalytic protocol was also suitable for the functionalization of olefins such as **3a** (Scheme 2b). Thus, **4a** could be obtained with good yield as a unique Z-isomer proving the high selectivity of the TAM with respect to other *ortho*-orientating DGs, used in iron-catalyzed C–H arylations of alkenes [41].

**Scheme 3:**
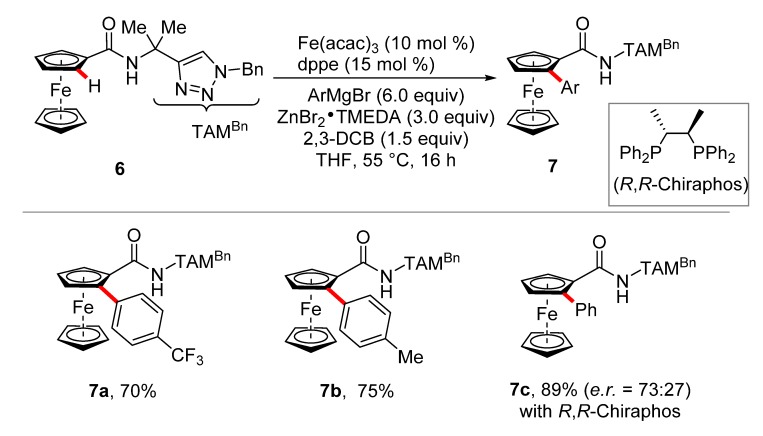
Iron-catalyzed C(sp^2^)–H arylation of ferrocenes aromatic rings with TAM DG.

The deprotection of TAM DG was carried out under acidic conditions, leading to *ortho*-arylated benzoic acid **5b** in high yields (Scheme 2c). TAM DG was found applicable for C–H arylations of ferrocene derivatives **6** as well [42]. Again, the combination of diphosphine ligand dppe and 2,3-dichlorobutane (2,3-DCB) as external oxidant was found essential for the transformation. The use of an in situ-formed Ar_2_Zn•MgBr_2_(TMEDA) organometallic base allowed for the conversion of amides **6a-b** into racemic products **7a-b** in good yields. Noteworthy, the use of a chiral ligand such as *R*,*R*-chiraphos led to the formation of a planar chiral ferrocene derivative **7c** with moderate enantiomeric ratio (Scheme 3).

**Scheme 4:**
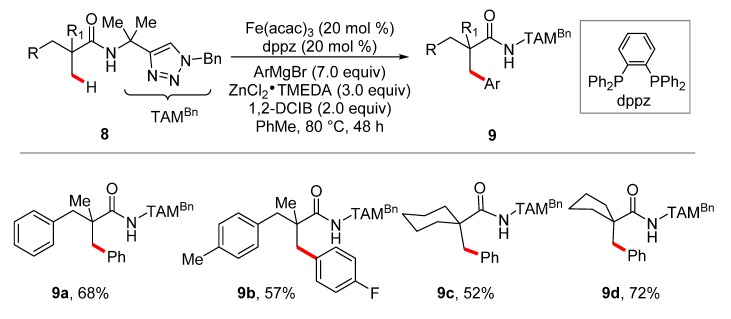
Iron-catalyzed C(sp^3^)–H arylation with TAM DG.

The activation of C(sp^3^)–H is generally more challenging than C(sp^2^)–H due to their lower acidity [43,44]. In analogy with previous reports on iron-catalyzed C(sp^3^)–H arylations enabled by 8-AQ DGs, TAM DG was found operative under similar conditions [40,45]. A broad range of substrates **8** was functionalized in good yields with a complete selectivity of the primary over the secondary C–H bond, rendering an organometallic C–H activation mechanism likely to be operative under present conditions (Scheme 4).

**Scheme 5:**
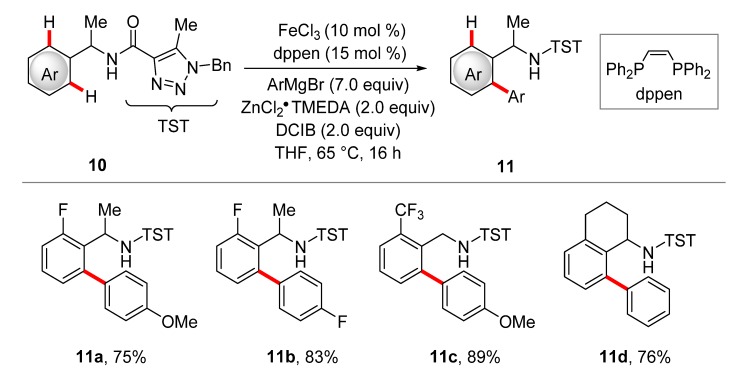
Iron-catalyzed C(sp^2^)–H arylations of benzylamides with tri-substituted triazole (TST) DG.

In a parallel fashion, the development of TST (tri-substituted triazole) DG opened access to C–H arylations of geometrically flexible, electron-rich benzylamide derivatives [46]. Here, a phosphine with a rigid back-bone, such as *cis*-1,2-bis(diphenylphosphino)ethene (dppen), was the key to efficiently functionalize benzylamides **10** in the presence of 1,2-DCIB as the mild oxidizing reagent (Scheme 5).

**Scheme 6:**
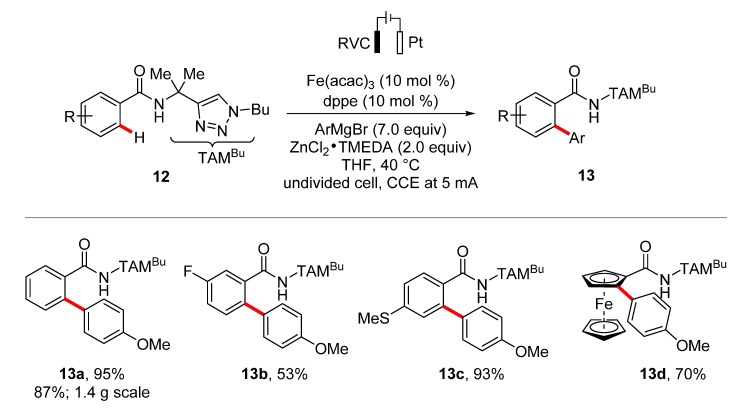
Electrochemical iron-catalyzed C(sp^2^)–H arylation with TAM DG.

Ackermann and co-workers recently explored, by computational analysis, the oxidation potential of the iron(II)/(III) manifold, revealing a viable redox event at E_1/2_ = 0.01 V (*vs* Fc^0/+^) that could be exploited under electrochemical conditions [47]. Interestingly, electrochemical C–H arylations of TAM amides **12** were feasible at an exceedingly mild reaction temperature of 40 °C, when using a reticulated vitreous carbon (RVC) anode, along with a platinum cathode (Scheme 6).

Hence, the dichloroisobutane (DCIB) sacrificial oxidant could be replaced by the introduction of the electrochemical oxidation. The robustness of the methodology was proved by the synthesis of oxidation-sensitive thioether **13c** and the electrochemical active ferrocene **13d**, which were perfectly tolerated, as well as the gram-scale reaction for the synthesis of **13a**. The performance of the heterogenous ferraelectrocatalysis was analyzed with respect to the homogeneous DCIB-mediated protocol. Indeed, the electrochemical oxidant-free approach outcompeted the chemical oxidant one in terms of chemical yields and kinetic profile.

**Scheme 7:**
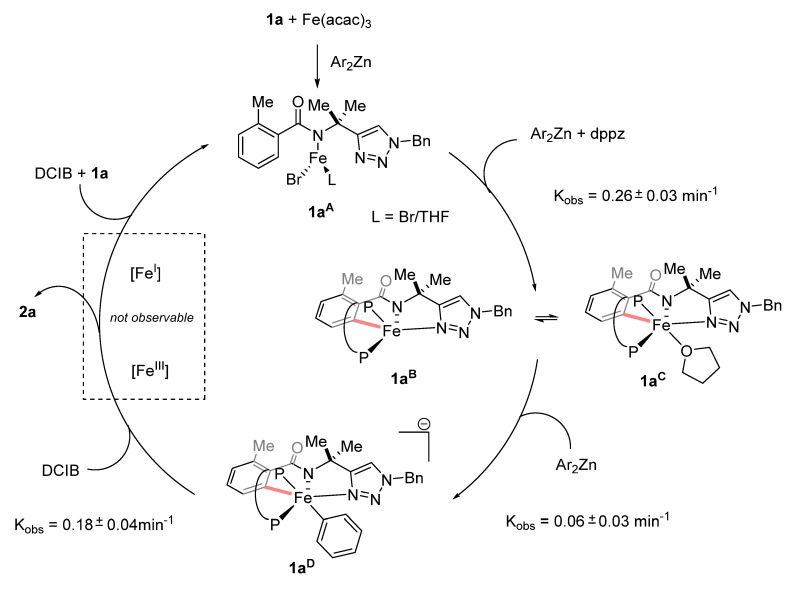
Proposed mechanism for iron-catalyzed C(sp^2^)–H bond arylation with TAM DG.

Very recently, mechanistic studies performed by Neidig and co-workers finally shed some light on the mechanism operating in these transformations, by the identification of key cyclometallated iron(II) intermediates present in triazole-directed C–H arylations [48]. Particularly, substrate **1a** was treated with 1 equiv of Ar_2_Zn reagent in the presence of Fe(acac)_3_ and 1,2-Bis(diphenylphosphino)benzene (dppz), leading to the predominant formation of a high-spin iron(II) species with ^57^Fe Mössbauer parameters of *δ* = 0.94 mm/s and ∆E_Q_ = 3.14 mm/s. Further studies conducted with magnetic circular dichroism (MCD) enabled the assignment of this species as a distorted, tetrahedral complex of type **1a^A^**. An analogous reaction performed with 3 equiv of organozinc reagent led to the consumption of intermediate **1a^A^** with the formation of two new low-spin iron species with Mössbauer parameters of *δ* = 0.30 mm/s and ∆E_Q_ = 1.92 mm/s (60%) and *δ* = 0.24 mm/s and ∆E_Q_ = 1.19 mm/s (17%), respectively. Further experimental work led to the identification of the major species as a cyclometallated iron(II) dimer [(**1a**)(dppbz)(THF)Fe]2(μ-MgX2) **1a^C^** by X-ray spectroscopy, in which a magnesium salt bridges two neutral monomers. The minor species was assigned to the five-coordinate, non-THF-ligated analogue **1a^B^**. To determine the rate of the formation of these two species, pseudo-single turnover studies using time-resolved, freeze quenched ^57^Fe Mössbauer spectroscopy were employed. Hence, a rate constant of 0.26 ± 0.03 min^−1^ via a pseudo first-order kinetic fit suggested a facile C–H activation. Furthermore, the stoichiometric addition of two more equivalents of Ar_2_Zn to a THF solution of **1a^B/C^** led to the formation of a new species, with Mössbauer parameters δ = 0.15 mm/s and ∆E_Q_ = 0.54 mm/s, which was indirectly identified with a cyclometallated low-spin iron(II)−phenyl dimer, [(**1a**)-(dppz)(Ph)Fe]_2_(μ-Mg(THF)_3_) **1a^D^**. Interestingly, this intermediate was found to be competent for C–C bond formation. Hence, its consumption to give the *ortho*-arylated product by stoichiometric reaction with DCIB was estimated again by freeze-trapped, time-resolved ^57^Fe Mössbauer studies to occur at an observed rate constant of 0.18 ± 0.04 min^−1^ under pseudo-first-order conditions. In this scenario, the role of DCIB was crucial; it promoted the oxidation of **1a^D^** via single-electron type (SET) mechanism to a putative iron(III) intermediate that subsequently undergoes reductive elimination to give the corresponding *ortho*-functionalized product **2a** and lower-valent iron(I) species. This finally re-enters the catalytic cycle after oxidation operated by a second equivalent of dihaloalkane (Scheme 7).

## 3. Iron-Catalyzed C–H Alkylations/Allylations

**Scheme 8:**
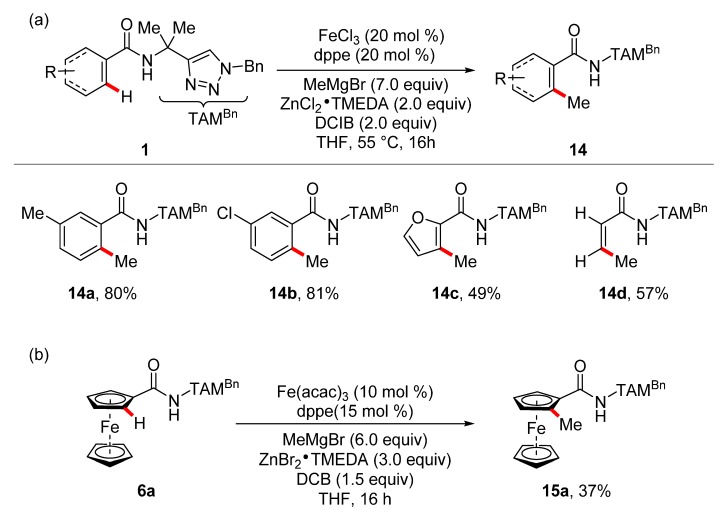
Triazole-assisted methylation of C(sp^2^)–H bond; (**a**) Selected examples of aryl amides; (**b**) C(sp^2^)–H methylation of ferrocene.

The development of atom- and step-economical strategies for the direct installation of medicinally relevant methyl groups are of high interest [49,50]. In this context, in 2015, Ackermann and co-workers reported the first example of iron-catalyzed methylation under triazole assistance [51]. It is noteworthy, that copper, palladium, and cobalt were ineffective in C–H methylations under otherwise identical reaction conditions, proving the advantage of iron catalysis [52]. The inexpensive FeCl_3_ catalyst and dppe as ligand enabled this transformation in the presence of an in situ-generated dimethyl zinc species and DCIB as mild oxidizing reagent. The TAM-assisted functionalization proved widely applicable for the activation of C(sp^2^)–H bonds of various aromatic TAM-amides **1**, with high yields and site selectivity (Scheme 8a). The procedure was not confined to aromatic C–H bonds but also enabled the activation of alkenyl C(sp^2^)–H bonds **14d** with good yield. In particular, the sole thermodynamically less stable *Z*-alkene was isolated, highlighting the fundamental triazole activity. In analogy with the arylation procedure, in 2017, Butenschön and co-workers achieved the methylation of ferrocene derivative **6a** in moderate yields, using analogous reaction conditions (Scheme 8b) [42].

**Scheme 9:**
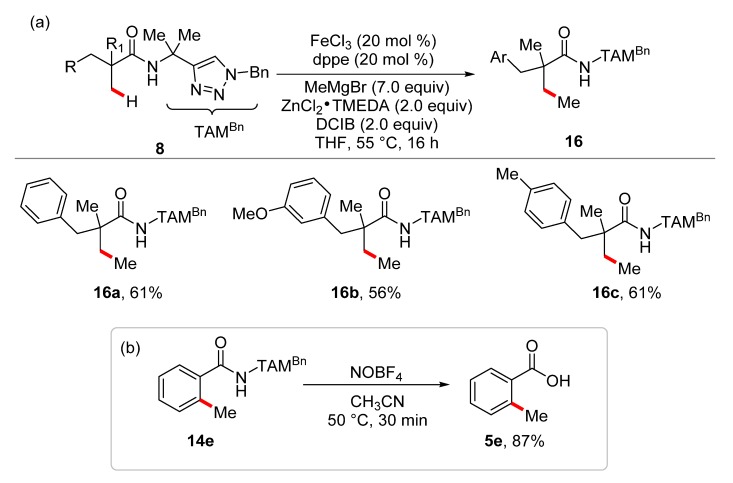
(**a**) Triazole-assisted methylation of amides; (**b**) Mild triazole removal to 2-methyl benzoic acid **5e**.

Challenging C(sp^3^)–H bonds were found to be suitable for the transformation as well; hence, Ackermann and co-workers exploited the high versatility of the triazole protocol showing C–H methylations to be applicable for the synthesis of various aliphatic amides **16a-c** (Scheme 9a). Finally, a complementary DG removal procedure was reported, leading to the *ortho*-methylated benzoic acid **5e** in high yields under remarkably mild reaction conditions (Scheme 9b).

**Scheme 10:**
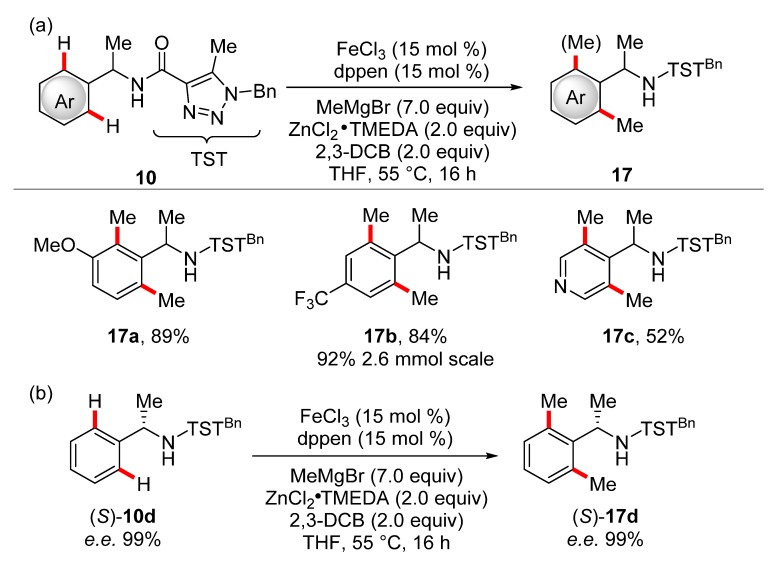
Iron-catalyzed triazole-assisted methylation of C(sp^2^)–H bonds; (**a**) Methylation of benzylamides **17a-c**; (**b**) Methylation of the enantioenriched amine **(*S*)-10d**.

Due to the importance of the methyl group in medicinal chemistry, the protocol was extended to pharmacologically relevant benzylamides derivatives [46]. The trisubstituted triazole TST DG was found essential in promoting C–H methylations with high levels of chemoselectivity and positional selectivity for a broad family of benzylamides **10** (Scheme 10a).

**Scheme 11:**
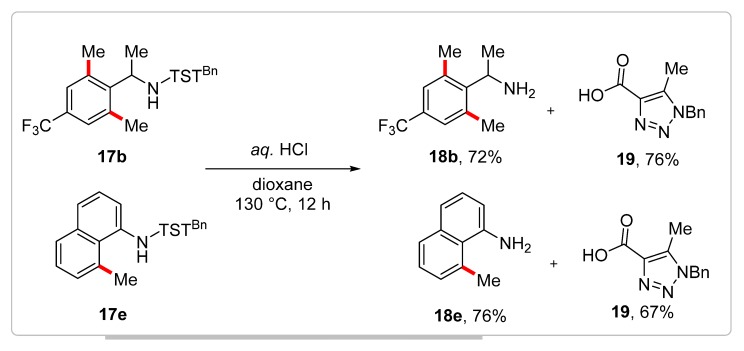
Removal and recovery of TST DG under acidic conditions.

Differently from the C–H arylation protocol and for reason yet to be clarified, here 2,3-DCB outcompeted 1,2-DCIB as the oxidant. The synthetic utility of the methodology was, among others, reflected by the racemization-free protocol for the functionalization of enantiomerically-rich benzamides (*S*)-**10d** (Scheme 10b). Finally, aqueous acidic conditions led to the deprotection of desired amines **18b**,**e** and allowed for the recovery of the reusable TST directing group **19**, in high yields (Scheme 11).

Intermolecular competition experiments demonstrated the highest reactivity of triazole DG TST compared to the picolinamide DG [31]. In sharp contrast with all the other methodologies described for oxidative iron-catalyzed C–H functionalizations, here a kinetic isotopic effect of *k*_H_/*k*_D_ = 1.8 was revealed. This suggested a kinetical relevant C–H activation step as a consequence of the inherent flexibility of substrates **10** that makes the cyclometallation challenging to occur.

**Scheme 12:**
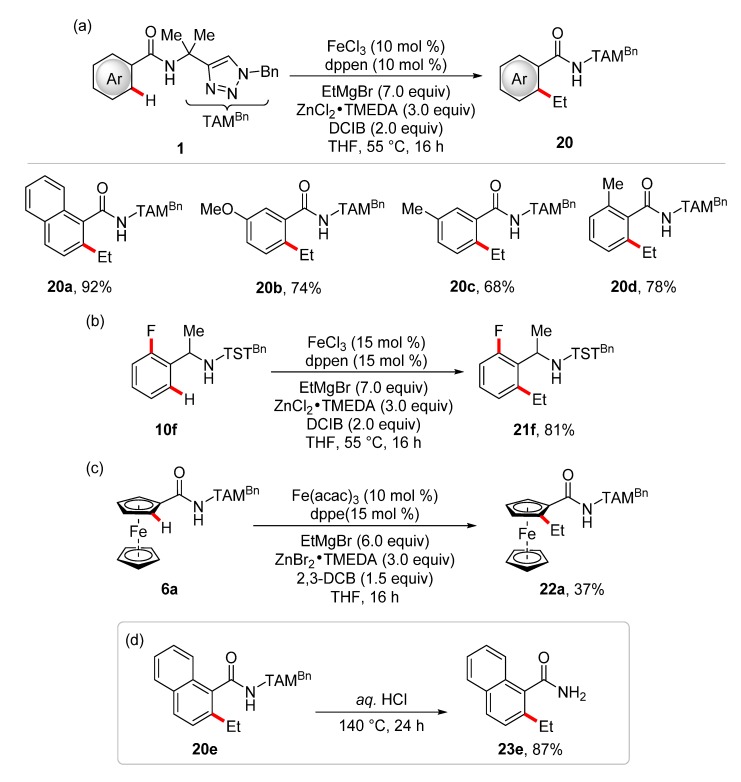
Iron-catalyzed triazole assisted ethylation of C(sp^2^)–H bond. (**a**) Ethylation of TAM amides **1**; (**b**) Ethylation of TST amide **10f**; (**c**) Ethylation of ferrocene amide **6a**; (**d**) Triazole removal protocol.

Oxidative conditions were found eligible for triazole-assisted, iron-catalyzed C–H ethylation, as well [42,46,51]. In this regard, Ackermann and co-workers reported C–H ethylations of benzamides **1** enabled by TAM that proceeded under oxidative conditions with 1,2-DCIB as the oxidant (Scheme 12a), whereas in 2017, the same group extended this methodology for the synthesis of benzylamine derivative **21f**, exploiting TST DG (Scheme 12b). In analogy with previous studies, Butenschön and co-workers reported the ethylation of challenging ferrocene derivative **6a**, albeit in lower yields (Scheme 12c). Surprisingly, in the case of **20e**, the removal of the TAM directing group under acidic conditions allowed to recover the *ortho*-functionalized primary amide **23e** in high yields. (Scheme 12d).

**Scheme 13:**
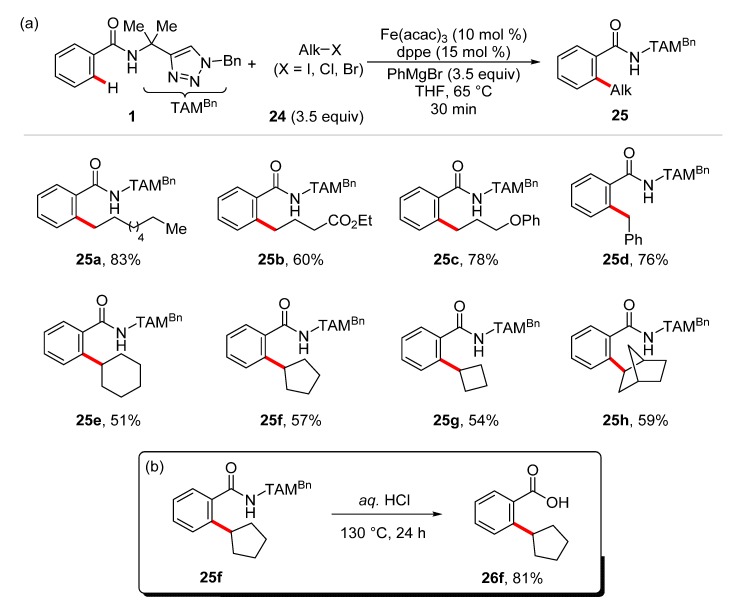
(**a**) Iron-catalyzed, TAM-assisted alkylation of C(sp^2^)–H bond; (**b**) TAM removal.

In 2016, Ackermann and co-workers reported the alkylation of aromatic C(sp^2^)–H bond using alkyl halides enabled by TAM-assisted iron catalysis [53]. Fe(acac)_3_ pre-catalyst and dppe as the ligand, provided the formation of new C–C bonds using commercially available primary and secondary alkyl bromides **24** [54,55]. The dropwise addition of the Grignard reagent allowed the reaction to proceed smoothly at a remarkable reaction time of 30 min, leading to the synthesis of *ortho*-functionalized benzoic acids derivatives **25** in high yields and functional-group tolerance (Scheme 13a). Interestingly, the AQ was found inactive under these reaction conditions. Moreover, iodomethane and benzyl chloride were also found suitable for C–H methylations and benzylations, highlighting the ample synthetical applicability of the catalytic methodology.

Hence, traceless removal of the TAM DG furnished 2-cyclopentylbenzoic acid **26f** from its parental amide **25f** in good yields under aqueous acidic conditions (Scheme 13b).

**Scheme 14:**
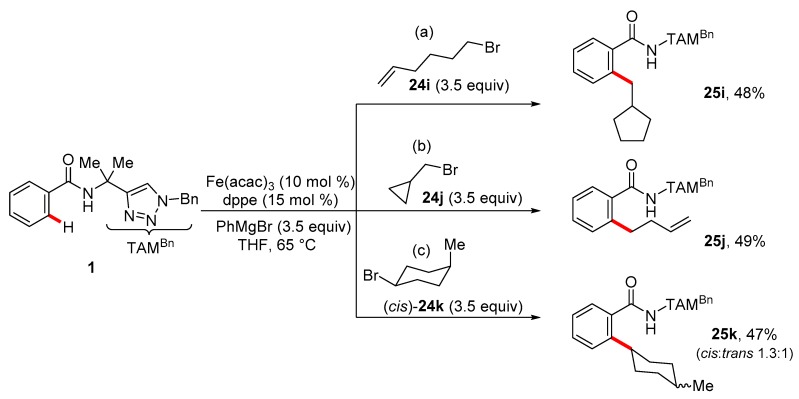
Radical mechanistic finding in iron-catalyzed alkylation of C(sp^2^)–H bond with (**a**) 6-bromohex-1-ene **24i**, (**b**) cyclopropylmethyl bromide **24j**, (**c**) *cis*-1-bromo-4-methylcyclohexane **24k**.

To probe the mechanism, iron-catalyzed C–H alkylations were performed with 6-bromohex-1-ene **24i** and cyclopropylmethyl bromide **24j** (Scheme 14a,b).

Hence, cyclopentylmethyl product **25i** and the linear alkene **25j** were delivered, respectively. In addition, complete racemization was observed in the product **25k** that was obtained starting from the diasteromerically pure *cis*-1-bromo-4-methylcyclohexane **24k** (Scheme 14c). Additionally, a considerable reduction of the conversion was also reported in presence of stoichiometric amounts of the radical scavenger TEMPO [(2,2,6,6-Tetramethylpiperidin-1-yl)oxyl]. All these observations suggested a single-electron-transfer (SET)-type mechanism to be operative [56].

**Scheme 15:**
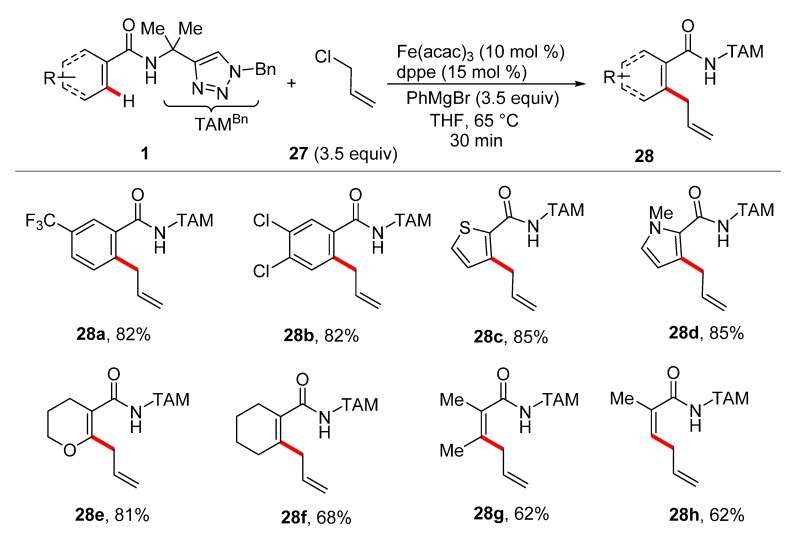
Iron-catalyzed TAM-assisted allylation of C(sp^2^)–H bond with allylchloride **27**.

The optimized catalytic system was subsequently tested for *ortho*-allylation of carboxamide derivatives **1**. Under these conditions, allyl chlorides **27**, which were previously proved ineffective using the 8-AQ DG [57,58], could be efficiently employed. High yields and regioselectivities were obtained for a broad family of amides **1** under oxidant-free conditions using Fe(acac)_3_ as the iron precursor and dppe as the ligand (Scheme 15).

As for C–H alkylations, dropwise addition of phenylmagnesium bromide was necessary for the reactivity. C(sp^2^)–H bond allylations were demonstrated to be applicable not only to functionalize aromatic and heteroaromatic rings but also for the synthesis of *ortho*-allylated alkenes **28e-h**. In this case, *Z*-alkenes stereoisomers were obtained with high yields and regioselectivity.

**Scheme 16:**
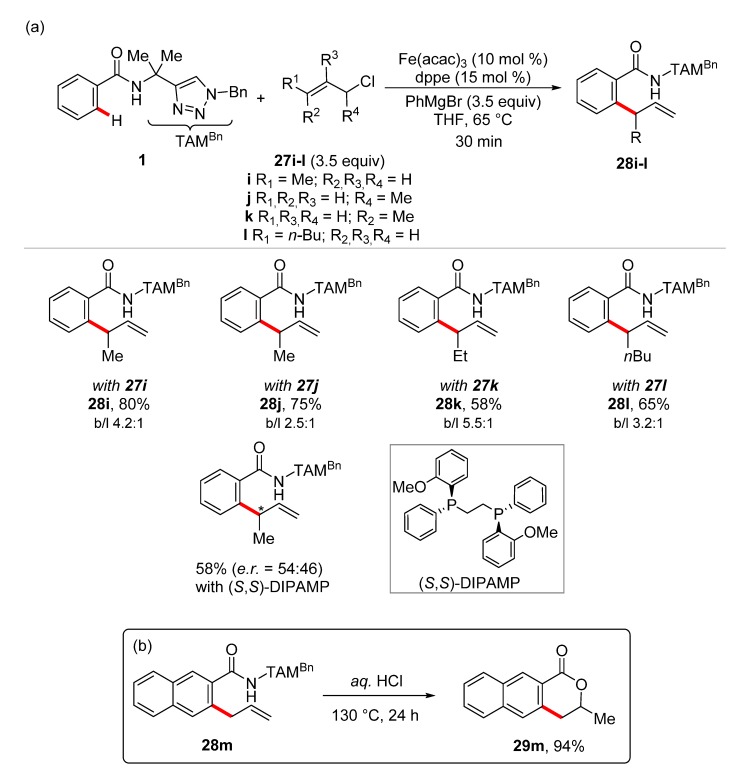
(**a**) Iron-catalyzed TAM-assisted allylation of C(sp^2^)–H bond with allylchlorides **27i-l**; (**b**) Isochromanone synthesis via TAM-deprotection.

The TAM DG proved its versatility allowing the use of substituted allyl chlorides **27i-l**. Intriguingly, the reaction with *E*-crotyl chloride **27i** and α-methyl allyl chloride **27j** yielded the same product **28i** with comparable regioselectivity (Scheme 16a). These results might suggest the presence of *η*^3^-allyl iron intermediates. Moreover, the regioselectivity associated to the formation of the branched product seemed to be dictated by the bidentate nature of the ligand, dppe, as observed in precedent studies by Plietker and co-workers [59,60,61]. This influence was further demonstrated conducting the reaction in the presence of a chiral ligand, such as commercially available (*S*,*S*)-DIPAMP. Under these conditions, **28i** could be isolated with a low, yet reproducible enantiomeric ratio; this result prompted subsequent studies on enantioselective iron-catalyzed C–H alkylations [62]. It is worth noting that the usefulness of the methodology was further revealed by the deprotection/functionalization of *ortho*-allylated benzamide derivative **28m** that selectively formed naturally-occurring isochromanone synthon **29m** in high yields (Scheme 16b).

Mechanistically, competition experiments with isotopically labelled substrates showed a kinetic isotope effect of k_H_/k_D_ 1.8 and 2.1 for intra- and intermolecular reactions, respectively. These results are coherent with a C–H bond activation step as rate determining in the reaction. Finally, a radical reaction pathway was excluded since the addition of radical scavengers did not affect the outcome of the catalysis.

## 4. Iron-Catalyzed C–H Functionalizations with Alkynes and Allenes

**Scheme 17:**
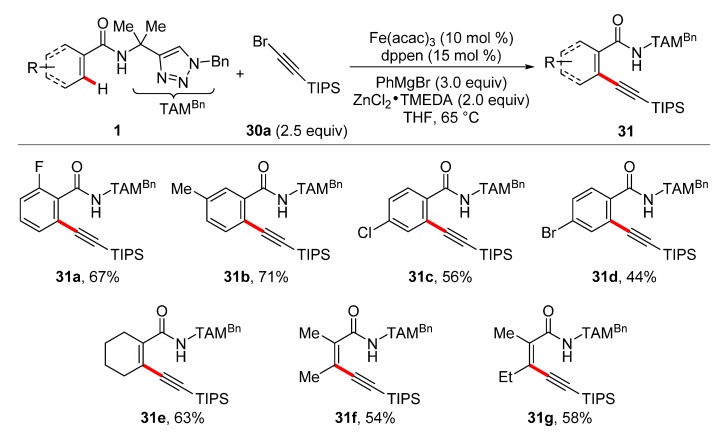
Iron-catalyzed TAM-assisted alkynylation of C(sp^2^)–H bonds with bromoalkyne **30**.

The alkyne fragment is one of the most versatile structures in organic synthesis; for this reason, synthetic and catalytic sustainable methodologies are constantly desired for their introduction in complex scaffolds. Transition metals-catalyzed alkynylation, often achieved through the well-established Sonogashira reaction, represents a strategic approach for the synthesis of alkynes derivatives [63,64,65]. In the context of C–H bond functionalizations [66], Ackermann and co-workers reported in 2017 the first iron-catalyzed C–H alkynylation using TIPS-bromoalkyne derivates **30a** [67]. The reaction proceeded with an iron(III) source as the pre-catalyst and a rigid dppen ligand, in presence of an organozinc base of type PhZnCl•MgBrCl(TMEDA) that was mandatory for the transformation. The TAM DG allowed the synthesis of a family of *ortho*-alkynylated carboxamides **31** with high yields and site selectivity (Scheme 17).

**Scheme 18:**
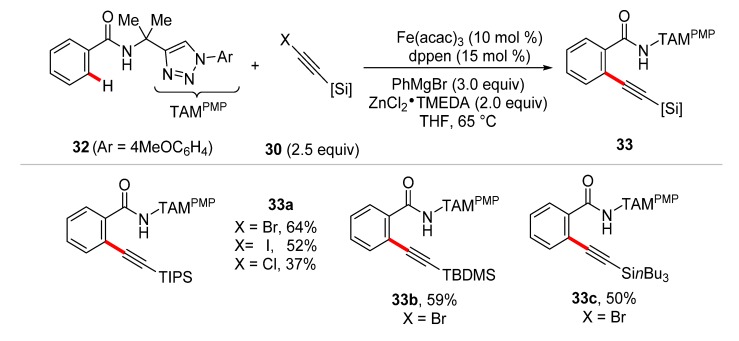
Probing iron-catalyzed C(sp^2^)–H alkynylation with haloakynes **30**.

Moreover, the reactivity of different haloalkynes **30** was tested employing the TAM^PMP^ DG; indeed, the supremacy of the bromide leaving group in iron-catalyzed C–H alkynylations was highlighted (Scheme 18).

**Scheme 19:**
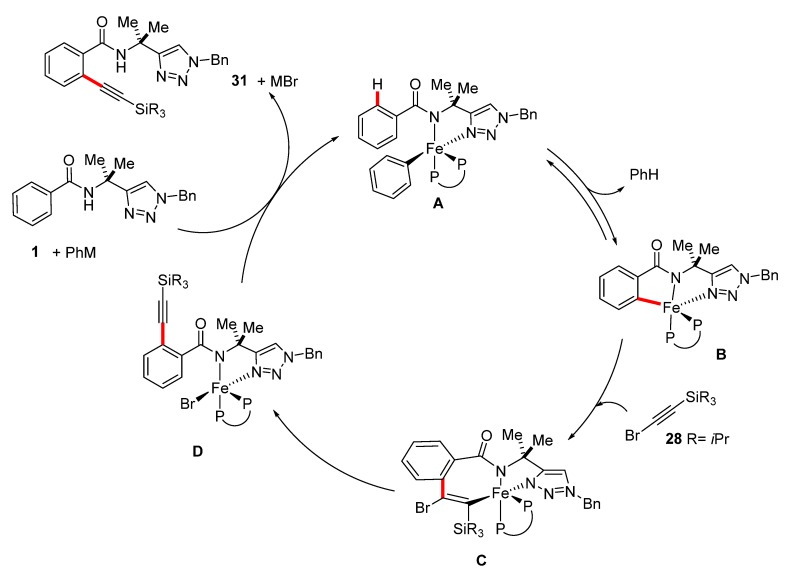
Proposed mechanism of iron-catalyzed TAM-assisted C–H alkynylation of amides.

Mechanistic investigations were reported; the use of radical scavengers led only to a slight reduction of the yields, excluding a single-electron-transfer mechanism to be operative. The superior reactivity of electron-poor arenes, with respect to the corresponding electron-rich, in intermolecular competition experiments suggested a deprotonative σ-bond metathesis (DBM) as the C–H activation step. In addition, cyclometallation was proposed to be not rate determining as indicated by a low kinetic isotopic effect of *k*_H_/*k*_D_ = 1.1. On the basis of these experimental results, the authors proposed a plausible catalytic cycle (Scheme 19). Organometallic iron species **A**, formed through coordination of iron pre-catalyst with amide **1** in the presence of organozinc reagent, was converted into **B** through a reversable C–H bond activation. A subsequent bromo-alkyne regioselective insertion provided metallacycle **C** that finally released product **31** and the active catalytic species via β−Br-elimination.

**Scheme 20:**
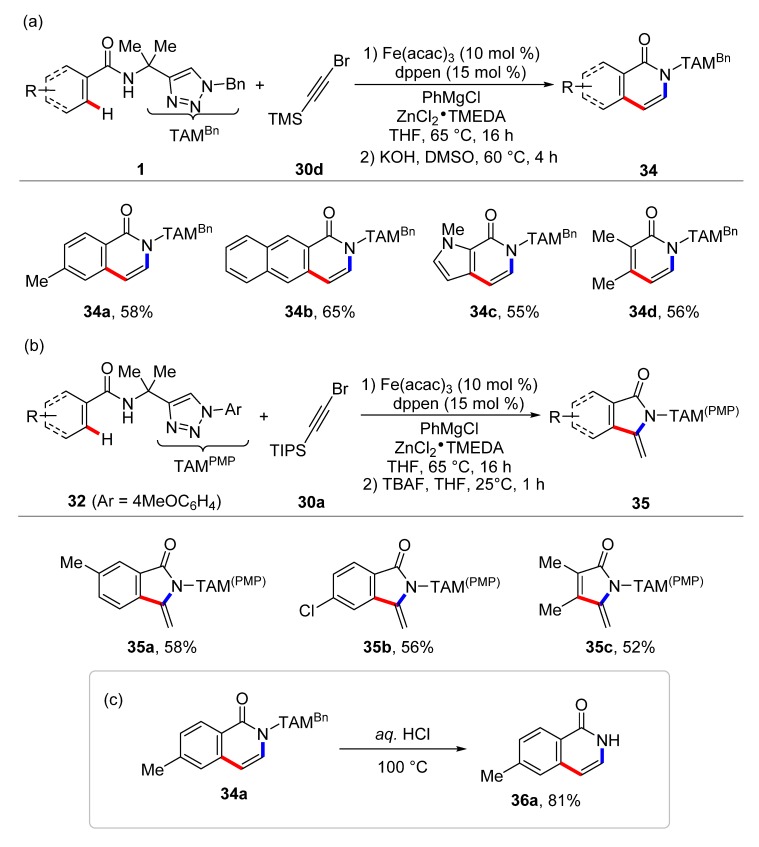
Iron-catalyzed C–H alkynylation/annulation. (**a**) Isoquinolones synthesis; (**b**) Isoindolinones synthesis; (**c**) Removal of TAM DG.

The use of alkynes in C–H functionalization methodologies, could be exploited for the synthesis of bioactive heterocycles. Among these molecules, isoquinolones have an interesting bicyclic structure that is present in multiple natural products and often used as a synthetic building block. Pictet–Spengler and Bischler–Napieralski reactions are the common strategies for the synthesis of these naturally occurring scaffolds. However, these methodologies require the use of pre-functionalized substrates [68]. Interestingly, by the interplay of reaction conditions, the triazole-assisted, iron-catalyzed C–H alkynylation reaction offered the opportunity to synthesize isoquinolone and isoindolinones in a sustainable, efficient manner [67]. Indeed, a one-pot/two-step cascade C–H alkynylation/6-*endo*-dig annulation proceeded smoothly with TMS-bromoalkyne **30d** and allowed for the synthesis of isoquinolone **33** after basic deprotection of TMS group (Scheme 20a). Parallelly, when *ortho*-alkynylated benzamides **33**, obtained by C–H alkynylation of **30**, were submitted to typical desilylative conditions, a 5-*exo*-dig annulation provided isoindolinones **34.** This class of molecules was obtained with excellent site- and chemoselectivity, and the protocol was found suitable for the synthesis of pyrrolone **35c** as well (Scheme 20b). Notably, NH-free isoquinolone **36a** could be isolated in high yields by TAM removal under acidic conditions (Scheme 20c).

**Scheme 21:**
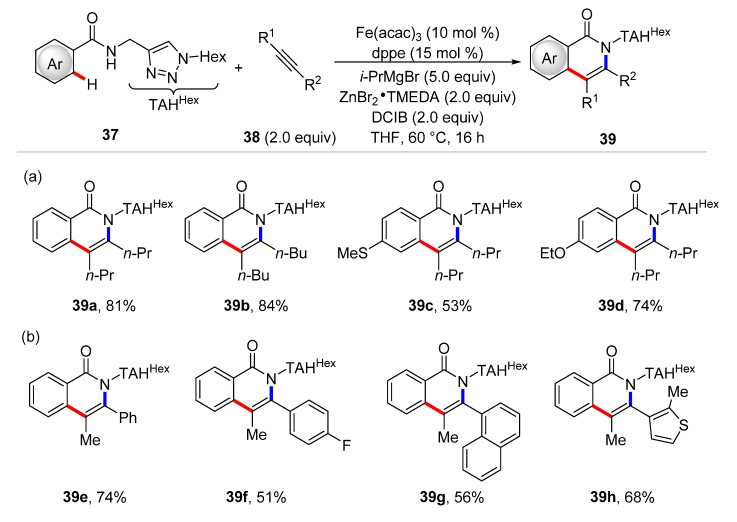
Iron-catalyzed C–H annulation of triazolyldihydrogenmethyl (TAH) amides. (**a**) Symmetric internal alkynes; (**b**) Methyl-aryl alkynes.

In order to devise a complementary approach for the synthesis of isoquinolones, Ackermann and co-workers probed TAM benzamides **1** for iron-catalyzed C–H annulation with alkynes **38** [69]. Interestingly, they always observed a lack of reactivity, leading to hypothesize an important contribution of the Thorpe–Ingold effect on the catalytic manifold [70]. Hence, the introduction of a new triazole TAH (triazolyldihydrogenmethyl) DG, being devoid of the *gem*-disubstitution, was demonstrated to be necessary for the synthesis of decorated isoquinolones **39** [71]. The use of an in situ-formed iPr_2_Zn•MgBr_2_(TMEDA) organometallic base was mandatory to promote the catalytic annulation with alkynes, along with the use of 1,2-DCIB as the oxidant. A broad range of TAH-benzamides **37** and internal alkynes **38** was suitable for the annulation, providing isoquinolone products **39a-d** with good yields (Scheme 21a). Disappointingly, for reasons yet to be rationalized, *meta*-substituents depleted the reactivity of the iron catalysis. The C–H annulation with alkynes proceeded with complete *anti*-Markovnikov regioselectivity for a broad range of methyl-aryl alkynes, delivering isoquinolones **39e**-**h** (Scheme 21b) in good yields. The regioselectivity observed in this process might be ascribed to the compact nature of the iron catalyst that influenced the coordination geometry of the alkyne in the key migratory insertion step.

**Scheme 22:**
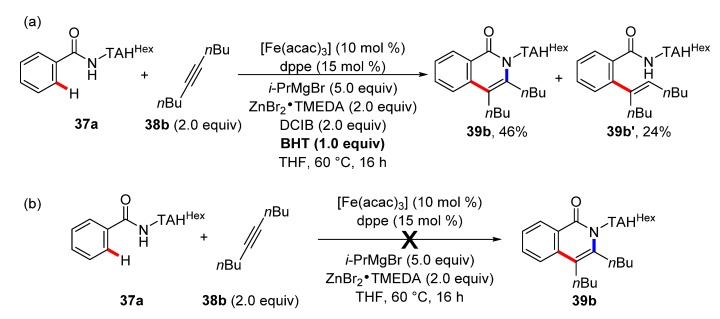
Mechanistic experiments for iron-catalyzed C–H annulations with alkynes; (**a**) Reaction conduced in presence of BHT as radical scavenger; (**b**) Stoichiometric reaction in absence of oxidant.

The mechanism was subsequently probed with several experiments. The reaction performed in the presence of radical scavengers led to a mixture of **39b** and the hydroarylated product **39b’** as the side-product. This latter, resubmitted to the optimized reaction conditions did not convert to the desired product **39b**, suggesting that a 7-membered metallacycle species represented a key intermediate in the reaction mechanism (Scheme 22a). As for previously illustrated C–H alkynylations, a kinetic isotopic effect as low as 1.1 suggested a C–H bond activation step not to be rate determining in the catalytic cycle. Finally, a stoichiometric reaction failed in delivering any product highlighting the crucial role of the dihaloalkane oxidant (Scheme 22b).

**Scheme 23:**
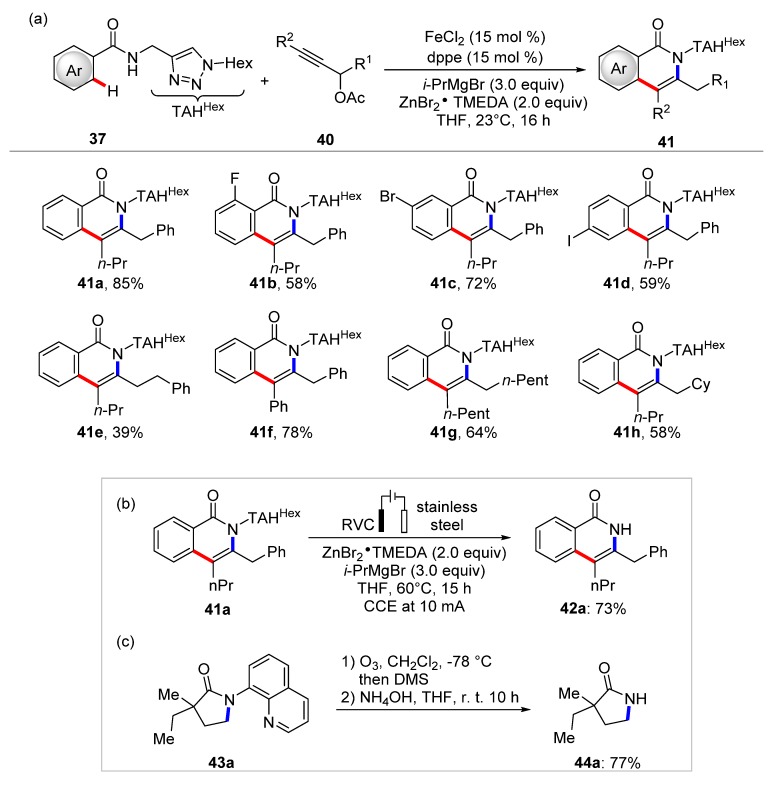
(**a**) Iron-catalyzed C–H annulation with propargyl acetate; (**b**) Electrochemical removal of TAH DG. (**c**) Oxidative cleavage of aminoquinoline (AQ) DG.

More recently, iron-catalyzed, triazole-assisted C–H functionalization methodologies have been exploited in the presence of propargyl acetates **40** for the synthesis of highly decorated isoquinolone derivatives [72]. As previously discussed, only TAH DGs were able to promote the highly selective C–H/N–H annulation reaction. In sharp contrast, various iron(II) and iron(III) pre-catalysts were demonstrated applicable for the catalysis, while only dppe ligand allowed the transformation to proceed in high yields. Notably, the catalysis occurred without the presence of an external oxidant. The versatile catalytic procedure led to the synthesis of a wide family of isoquinolones **41** in excellent to good yields and regioselectivities (Scheme 23a). The synthetic advantages were demonstrated by using different acetates and TAH-benzamides **37**, proving a high functional-group tolerance. The synthetic utility of the methodology was additionally demonstrated by a simple electrochemical cleavage of the TAH DG that delivered NH-free isoquinolone **42a** in high yield and chemoselectivity (Scheme 23b). This approach presented a notable advantage with respect to recent removal protocols of the AQ DG that required the use of hazardous chemicals such as ozone and dimethylsulfide (DMS) (Scheme 23c) [73].

**Scheme 24:**
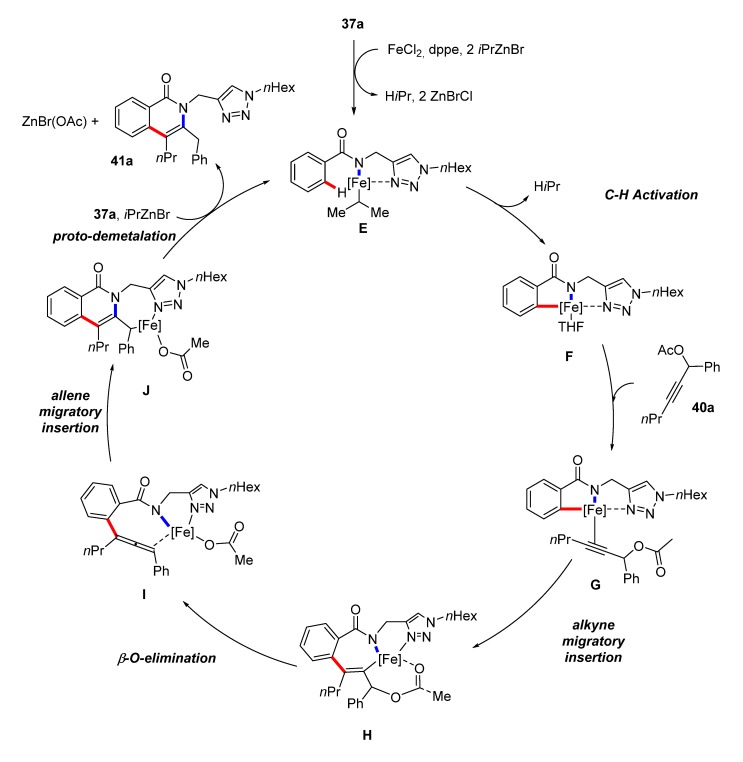
Proposed catalytic cycle for TAH-assisted C–H/N–H annulation with propargyl acetates.

Detailed mechanistic studies were conducted submitting deuterium-labelled benzamides **37** to the optimized reaction conditions. Hence, a considerable H/D exchange at the *ortho*-position was suggestive of a reversible C–H bond activation step. In addition, a kinetic isotope effect of k_H_/k_D_~1.1, excluded the C–H metalation as part of the rate-determining step. The mode of action of the C–H/N–H activation reaction was also investigated by DFT calculations taking in consideration all the possible iron(II) spin configurations. The lowest energy pathway was described by a sequence of four elementary steps: (1) C–H bond activation, (2) alkyne insertion, (3) β-O-elimination, and (4) allene migratory insertion. Subsequently, more detailed studies performed via Mössbauer spectroscopy [74] provided strong support for high-spin iron(II) intermediates [75], in sharp contrast to recent findings obtained for iron-catalyzed C–H arylations [48,63]. On the base of experimental and computational results, the authors proposed a catalytic cycle for the iron(II)-catalyzed C–H/N–H annulation sequence (Scheme 24).

Initially, the coordination of iron(II) to substrate **37a** in the presence of the organometallic base, led to intermediate **E**. Then, through a ligand-to-ligand hydrogen transfer (LLHT), metallacycle **F** could be formed [76]. After coordination of the ligand, a migratory insertion of the alkyne provided **H**, with the oxygen coordinated to the metal center, which subsequently underwent a C–O bond cleavage to deliver allenyl intermediate **I**. Insertion of the allene moiety into the N–Fe bond formed the annulated iron complex **J**, which finally released product **41a** through proto-demetalation with the regeneration of the catalytically active species **E**.

**Scheme 25:**
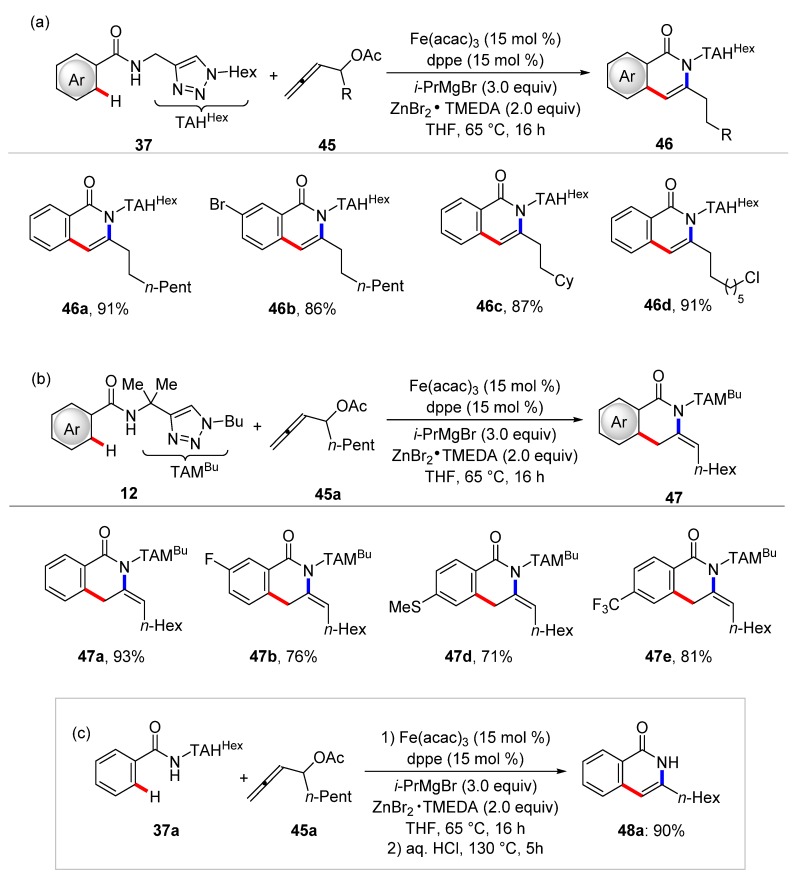
Iron-catalyzed C–H/N–H bonds activation/annulation with allenes; (**a**) TAH-assisted isoquinolones synthesis; (**b**) TAM-assisted dihydroisoquinolones synthesis; (**c**) One-pot/two-step isoquinolone **48a** synthesis.

Encouraged by the high catalytic activity demonstrated by iron catalysts along with triazoles and interested in the singular synthetic versatility of allene fragments [77], Ackermann and co-workers turned their attention to allenenyl acetates **45** as possible reagents for iron-catalyzed C–H/N–H annulation [78]. Indeed, the TAH-assisted annulation reaction within benzamides **37** proceeded smoothly in presence of Fe(acac)_3_ and dppe as the ligand. The use of an in situ-formed iPr_2_Zn•MgBr_2_(TMEDA) organometallic base was necessary, whereas, also in this case, no external oxidant was required. Noteworthy, for the first time, the modular electronic and steric properties of the triazole DG enabled complementary reactivities. As already observed, an important Thorpe–Ingold effect of methylene-tethered triazole moiety was responsible of the chemoselectivity associated to the transformation. Hence, under identical reaction conditions, a more flexible TAH DG provided the sole formation of isoquinolone products **46** (Scheme 25a), while the use of TAM DG, led to non-aromatic *exo*-methylene dihydroisoquinolones **47** (Scheme 25b). Finally, a one-pot/two-step protocol was realized for the selective synthesis of NH-free product **48a** in high yields (Scheme 25c).

**Scheme 26:**
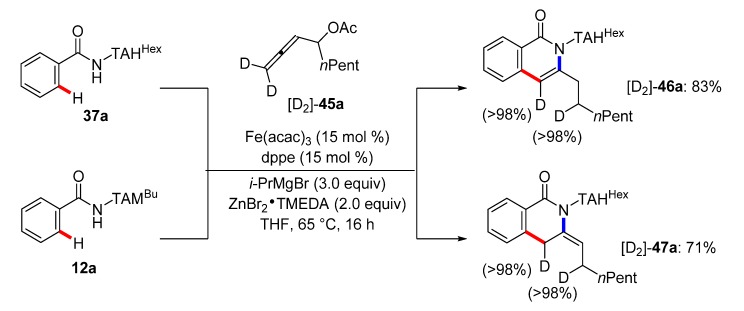
Mechanism investigation with isotopically labelled allene [D_2_]-**45a**.

To shed light on the reaction mechanism, intermolecular competition experiments were performed, and deuterium-labelled substrates were tested. The higher reactivity demonstrated by electron-deficient aromatic rings excluded a concerted-metalation-deprotonation (CMD) pathway [79,80]. Hence, that reactivity could be explained in terms of LLHT pathway or base-assisted internal electrophilic-type substitution (BIES) [81,82] Isotopically-labelled benzamides **37** shown a *k*_H_/*k*_D_ = 1.2 value proving a facile C–H bond cleavage that is therefore not involved in the rate-determining step. Under the standard conditions, the use of the *gem*-deuterated allene [D_2_]-**45a** in presence of TAH^Hex^ and TAM^Bu^ DGs, led to the formation of products [D_2_]-**46a** and [D_2_]-**47a**, respectively. Hence, a selective deuterium transposition was observed for both cases (Scheme 26).

**Scheme 27:**
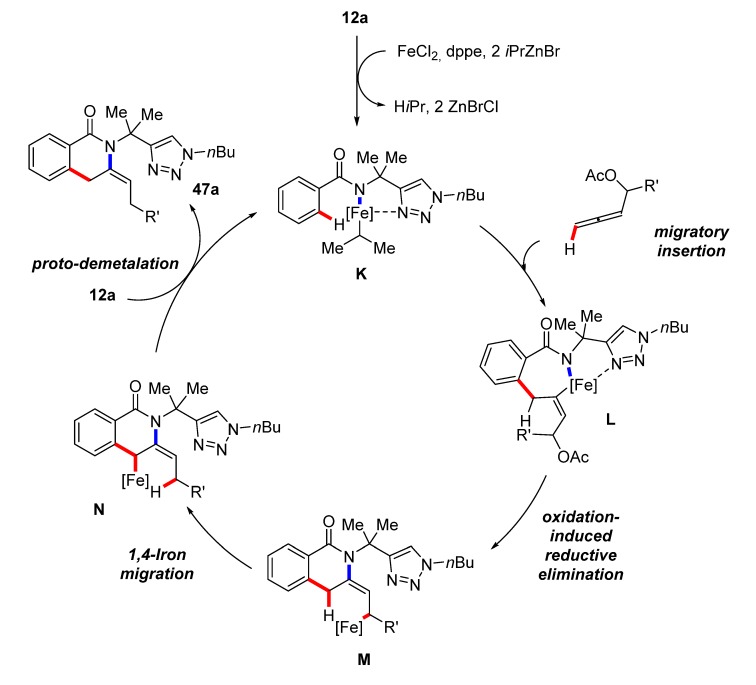
TAM-assisted C–H/N–H/C–O/C–H activation/annulation with allenes.

Based on these findings, the authors proposed a plausible mechanism pathway (Scheme 27).

The annulation reaction begins with a base-assisted coordination of the metal center to TAM-benzamide **12a**, forming intermediate **K**. Hence, a reversable C–H metalation followed by allene migratory insertion delivered cyclometalated species **L**. The latter could generate an η*^3^*-allyl-iron intermediate through an oxidative-induced reductive elimination to **M.** At this point, according to deuterium-labelling studies, a 1,4-iron migration eventually led to key intermediate **N**, that finally furnished the product via proto-demetallation, reforming the catalytic active specie **K**.

## 5. Conclusions

The development of sustainable 3d metals-catalyzed C–H bond functionalizations results nowadays as an important alternative to the use of toxic and expensive noble metals. In particular, the use of Earth-abundant, cheap, and non-toxic iron catalysts, renders organometallic C–H activation strategies the most user-friendly platform for the development of new catalytic methodologies for the constructions of C–C and C–Het bonds (for *ortho*-selective miscellaneous reactions, see [83,84]). In this context, the use of triazole-based *ortho*-orientating DGs enables complementary reactivities in iron-catalyzed C–H bond functionalizations, dictated by their modular geometry and electronics. Hence, C–H arylations, alkylations, and alkyne-annulations of amides and amines can be achieved with high yields and regioselectivity. In addition, simple and mild procedures for the introduction and removal of this DG result applicable for a wide range of functional groups. Recently, elegant experimental and computational studies shed some light on the valence and the spin state of catalytically active iron species, highlighting the crucial role played by phosphine ligands and triazole DG in controlling SET-type chemistry of organoiron compounds. This constitutes an important advance toward the design of more sustainable, organometallic reagent or eventually, DG-free catalytic systems. Furthermore, major efforts need to be directed toward the development of novel asymmetric C–H functionalization methodologies and heterogenous iron-catalyzed transformations, with the aim to impact this fascinating area of research.
